# *HGF*-rs12536657 and Ocular Biometric Parameters in Hyperopic Children, Emmetropic Adolescents, and Young Adults: A Multicenter Quantitative Trait Study

**DOI:** 10.1155/2019/7454250

**Published:** 2019-02-03

**Authors:** Jesús Barrio-Barrio, Elvira Bonet-Farriol, Marta Galdós, Susana Noval, Victoria Pueyo, Charles E. Breeze, Jose Luis Santos, Belén Alfonso-Bartolozzi, Sergio Recalde, Ana Patiño-García

**Affiliations:** ^1^Department of Ophthalmology, Clínica Universidad de Navarra, Navarra Institute for Health Research (IdiSNA), 31008 Pamplona, Spain; ^2^Department of Ophthalmology, Hospital de Cruces, 48903 Bilbao, Spain; ^3^Department of Ophthalmology, Hospital Universitario La Paz, Autonomous University, IdiPaz, 28046 Madrid, Spain; ^4^Aragon Institute for Health Research (IIS Aragón), Ophthalmology Department, Miguel Servet University Hospital, 50009 Zaragoza, Spain; ^5^UCL Cancer Institute, University College London, London WC1E 6BT, UK; ^6^Department of Nutrition, Diabetes and Metabolism, School of Medicine, Pontificia Universidad Católica de Chile, 8330077 Santiago, Chile; ^7^Department of Pediatrics and Clinical Genetics Unit, Clínica Universidad de Navarra, Navarra Institute for Health Research (IdiSNA), 31008 Pamplona, Spain

## Abstract

**Introduction:**

Even though ocular refractive state is highly heritable and under strong genetic control, the identification of susceptibility genes remains a challenge. Several *HGF* (hepatocyte growth factor) gene variants have been associated with ocular refractive errors and corneal pathology.

**Purpose:**

Here, we assess the association of an *HGF* gene variant, previously reported as associated with hyperopia, and ocular biometric parameters in a multicenter Spanish cohort.

**Methods:**

An observational prospective multicenter cross-sectional study was designed, including a total of 403 unrelated subjects comprising 188 hyperopic children (5 to 17 years) and 2 control groups: 52 emmetropic adolescents (13 to 17 years) and 163 emmetropic young adults (18 to 28 years). Each individual underwent a comprehensive eye examination including cycloplegic refraction, and topographic and ocular biometric analysis. Genomic DNA was extracted from oral swabs. *HGF* single nucleotide polymorphism (SNP) rs12536657 was genotyped. Genotypic, allelic, and logistic regression analyses were performed comparing the different groups. A quantitative trait association test analyzing several biometric parameters was also performed using generalized estimating equations (GEEs) adjusting for age and gender.

**Results:**

No association between rs12536657 and hyperopia was found through gender-adjusted logistic regression comparing the hyperopic children with either of the two control groups. Significant associations between mean topographic corneal curvature and rs12536657 for G/A (slope = +0.32; CI 95%: 0.04–0.60; *p*=0.023) and A/A (slope = +0.76; CI 95%: 0.12–1.40; *p*=0.020) genotypes were observed with the age- and gender-adjusted univariate GEE model. Both flat and steep corneal topographic meridians were also significantly associated with rs12536657 for the G/A and A/A genotypes. No association was found between rs12536657 and any other topographic or biometric measurements.

**Conclusions:**

Our results support a possible role for *HGF* gene variant rs12536657 in corneal curvature in our population. To our knowledge, this is the first multicenter quantitative trait association study of *HGF* genotypes and ocular biometric parameters comprising a pediatric cohort.

## 1. Introduction

Refractive errors are caused by a complex interaction between genetic and environmental factors, they are considered polygenic and multifactorial, and their etiology is not fully understood [1]. Nevertheless, the majority of the variance of refractive error within populations is thought to be due to hereditary factors [2]. In fact, the heritability of refractive errors has been estimated by several studies to be between 71% and 88% [3–5]. Although refractive state appears to be highly heritable and under strict genetic control, the identification of susceptibility genes until now has been challenging, with most studies focusing on myopia [6–10].

Hyperopia is the most common refractive error in childhood [11] and may be classified as low (<+2.00 diopters (D)), moderate (between +2.00 D and +5.00 D), or high (>+5.00 D). Among children, moderate and high hyperopes are a group of particular clinical relevance as significant hyperopia is clearly associated with some of the most frequent ocular conditions requiring multiple consultations at these ages, including mainly (but not only) strabismus and unilateral or bilateral amblyopia [12]. Levels of hyperopia between +3.00 and ≤+4.00 posed more than a 23-fold increase in esotropia risk compared to children with 0 to ≤+1.00 D of hyperopia in a population-based sample of 9970 children aged 6 to 72 months [13]. Hyperopia is also a major refractive risk factor for bilateral decreased visual acuity, the odds of which increased substantially for levels of hyperopia ≥+4.00 D [14]. Moderately hyperopic refraction has also been associated with astigmatism, anisometropia, abnormal convergence, reduced accommodative response, abnormal stereoacuity, self-reported eyestrain symptoms, and learning difficulties with poorer near visual performance with increasing hyperopia [15–17]. In fact, the popular belief that hyperopia diminishes with age appears not to be true for at least some hyperopic children who may have problems becoming emmetropic, and this is sometimes associated with an increase of accommodative lag [18–20]. Therefore, we wanted to focus our genetic study on moderate and high hyperopic school-age children, a population that represents a particularly vulnerable group for the conditions associated with hyperopia at these specific ages, which may also have effects on the learning process.

The *HGF* (hepatocyte growth factor) gene was one of the first candidate genes to be studied in relation to refractive errors and in fact the association of several single nucleotide polymorphisms (SNPs) of the *HGF* gene with myopia has been reported and replicated in independent studies on adults in Chinese [21, 22] and Caucasian populations [23, 24]. The first positive genetic association for hyperopia was published in 2010 when the association of SNPs rs12536657 and rs5745718 of the *HGF* gene with hyperopia was reported in a case-control study of the Australian population comprising emmetropic, hyperopic, and myopic adult subjects. These two SNPs showed strong linkage disequilibrium (LD) in the studied population [24]. Although recent genome-wide association studies (GWAS) have focused on other genes related to refractive state and hyperopia [25], the association of the *HGF* gene with hyperopia has not been studied in other populations. In addition, the *HGF* gene has been found to be associated not only with hyperopia but also with corneal pathology in several studies [26–28]. We therefore designed a study aiming to compare a group of hyperopic children to an emmetropic group. A specific problem when considering emmetropic children as a control group in genetic refractive error studies is the definition and frequency of childhood emmetropia. On the one hand, mild physiological hyperopia is the most common refractive state among neonates and infants [29, 30], even though the proportion of infants with hyperopia decreases by emmetropization from 6 months of age to a low point between 2 and 2.5 years of age across all ethnicities [31, 32]. Mild hyperopia seems to be the natural state of refractive development in children since the proportion of emmetropic (>−0.5 D but <+0.5 D) children between 5 and 15 years is scarce and in any case no more than 35% in any of several different sites around the world according to a large multiethnic study [33]. In fact, children with <+0.75 D or +0.50 D, depending on the age, are at increased risk of developing myopia in the future because of the growth of the eye [34]. Therefore, it is challenging to categorize these children as bona fide controls. Eye elongation decelerates in the second decade of life stagnating between 13 and 18 years [35]. Thus, we designed a study in which only older emmetropic children (13–17 years) and young emmetropic adults (18–28 years) were included as two separate control groups. Importantly, two factors support this design. Firstly, only adolescent and young adult controls present a bona fide set of nonmyopic subjects while still retaining childhood genotypic information. This is further supported by the impossibility of reverse causation in a genetic study, where the genotype cannot be affected by the phenotype [36]. Secondly, the specific statistical analyses employed are designed to account for any confounding age- and sex-related variation in this design.

Recently, several studies [6, 22, 37] have analyzed the refractive power of the eye as a continuous spectrum of refraction measurements instead of a binary trait (myopia versus hyperopia). Total ocular refractive power is thought to be modulated by a set of highly heritable underlying quantitative components (endophenotypes). These intermediate traits are thought to have a simpler genetic architecture and may be more sensitive measures of notable aspects of the disease process [5]. Quantitative trait association studies are ideally designed to analyze these endophenotypes. Recent GWAS has analyzed several endophenotypes: axial length [10], corneal curvature [38–42], and central curvature thickness [43]. Our main aim was to perform a quantitative trait study analyzing the association of *HGF* variant rs12536657 with biometric ocular measurements and several corneal parameters obtained by topographic analysis in a population of hyperopic and emmetropic children and emmetropic young adults.

## 2. Methods

### 2.1. Participants

An observational prospective multicenter cross-sectional study was designed. The study included 403 unrelated Spanish Caucasian subjects comprising 188 moderately or high hyperopic children aged 5 to 17, 52 emmetropic adolescents aged 13 to 17, and 163 emmetropic young adults aged 18 to 28. The patients were recruited between 2012 and 2015 at four Spanish hospitals: Clínica Universidad de Navarra (Pamplona), Hospital Universitario La Paz (Madrid), Instituto Clínico Quirúgico de Oftalmología (Bilbao), and Hospital Universitario Miguel Servet (Zaragoza).

The inclusion criteria for the emmetropic groups were patients aged 13 to 17 years (children emmetropic group) or 18 to 28 years (young adult emmetropic group), with an uncorrected monocular visual acuity (UCVA) of at least 1.0 (on the Snellen visual acuity scale) and a spherical equivalent (SE) of >−0.50 D <+1.25 in both eyes after cycloplegic refraction (cyclopentolate 1%). In the hyperopic children group, patients were aged 5 to 17 years, with hyperopia in both eyes with an SE of ≥+3.50 in the less hyperopic eye. Several reasons lead us to design our study groups with different ages: (1) the main target population of our study was the child population that suffers the consequences of the hyperopic condition; (2) the difficulty to find a bona fide control group of emmetropic young children (5 to 12 years) since emmetropia is an evolving process over the years and does not stabilize until the late teens or adulthood. On the other hand, moderate or high hyperopia present at these ages tends to have much less change, and therefore, its dioptric amount frequently remains nearly the same until adulthood; and (3) there are few studies on genetics comprising actual emmetropic young adults verified with cycloplegic refraction. Several measures were taken to adjust for any possible confounders in view of this specific design (explained in detail in the statistical analysis section).

The exclusion criteria included any ethnicity different from Spanish Caucasian, astigmatism exceeding 3.00 D, and any ocular conditions unrelated to the refractive error. Eyes with prior surgical history or low data quality were excluded. Children with systemic diseases were also excluded. Any individual with a family history of other eye diseases, such as high myopia, nanophthalmos, or keratoconus, was also excluded from the study. Individuals had to have an available DNA sample for inclusion in the study. All procedures were performed in accordance with the Declaration of Helsinki. All patients and caregivers received detailed information about the nature of the research and provided written informed consent before study enrolment. All of the local ethics committees of the participating centers approved the study.

### 2.2. Clinical Exam

All participants received comprehensive ophthalmic examination including best-corrected visual acuity, slit-lamp biomicroscopy of anterior segment, and retina with mydriasis. Cycloplegic autorefraction was assessed approximately 30 minutes after instillation of the last of 3 drops of 1% cyclopentolate given 5 minutes apart. Spherical equivalent (SE) was calculated as spherical error plus half the cylindrical error. Axial length, corneal curvature, white-to-white measurement, and anterior chamber depth were measured using an IOL Master 500® (software version 7.1; Carl Zeiss Meditec, Jena, Germany); the average of 5 measurements was taken into account for the analyses. Topographic analysis was performed with Sirius® (Phoenix software version 1.0.5.72; CSO, Florence, Italy). The topographic measurements analyzed included corneal curvature (SimK values: flat corneal meridian keratometry, steep corneal meridian keratometry, and mean keratometry), mean central corneal thickness, apical corneal thickness, white-to-white corneal diameter, anterior chamber angle, and mean anterior chamber depth. Tests were repeated until reliable measurements were obtained. Every test was performed in both eyes for each patient.

### 2.3. SNP Genotyping

Genomic DNA was extracted from oral swabs using QIAcube (Qiagen) in each of the participant centers. All the specimens were codified and sent to the Clínica Universidad de Navarra (Pamplona, Spain) where genotyping was performed by real-time PCR using TaqMan SNP genotyping assays (Applied Biosystems, Inc. (ABI), Foster City, CA) for SNPs rs12536657 of the *HGF* gene, following the manufacturer's instructions. An ABI 3730 real-time PCR machine (Applied Biosystems) was employed. Since the two SNPs of the *HGF* gene that were reported as associated with hyperopia (rs12536657 and rs5745718) showed strong linkage disequilibrium (LD), we decided to focus our analysis on the SNP rs12536657. Regarding the rationale for choosing HFG rs12536657 instead of rs5745718, we took into account several reasons: (1) rs12536657 was associated with low myopia and with hyperopia in the study by Veerappan et al. [24] and also with myopia in Yanovitch et al. [23], and therefore, it could be a stronger candidate for refractive error association and (2) in the study by Veerappan et al., when hyperopic and emmetropic groups were compared for rs12536657 under an additive model, each of the genotypes were found significant with an increasing odds ratio for each additional copy of the risk allele (OR: G/G: 1; G/A: 1.88; A/A: 5.53). In the case of rs5745718, only the C/A genotype was found to be associated with hyperopia [24].

### 2.4. Statistical Analysis

Genotype and allele frequencies for SNP rs12536657 were compared between different groups using the chi-squared test under an additive model. The comparison groups were hyperopic children (*n*=188) versus all the emmetropic patients (*n*=215); hyperopic children versus emmetropic children; and hyperopic children versus emmetropic adults. Likelihood-ratio tests were calculated to assess the agreement between observed genotype frequencies and Hardy–Weinberg equilibrium (HWE). All genome coordinates described in the text are from genome build hg19. Power calculations were performed using Quanto v1.2.4, considering a minor allele frequency of 0.14 (averaged from previously reported frequencies by Veerappan et al. [24]). Power calculations indicated that 178 individuals per group were needed to detect a minimum odds ratio (OR) of 1.8 with a power of 80% and an alpha error of 0.05 under an additive genetic model, assuming an equal sample size of cases and controls.

In addition, logistic regression was used for analysis of hyperopia as a qualitative trait for SNP rs12536657 and the same comparison groups. An additive genetic model was used in all regression tests. Therefore, one allele was assigned as the reference allele and the other the risk allele; the effect size per copy of minor allele was calculated for SNP rs12536657. Age and sex were included as additional covariates where appropriate, and conditional logistic regression was used to test if there was any association between age and genotypes.

The univariate generalized estimating equation (GEE) method was used to analyze the quantitative trait association of refractive measurements: spherical equivalent (SE) and astigmatism; IOL master biometric measurements: axial length, corneal curvature, and anterior chamber depth; and topographic measurements: corneal curvature, central corneal thickness, apical corneal thickness, horizontal corneal diameter (white-to-white corneal measurement), and anterior chamber depth under an additive genetic model. The age- and gender-adjusted GEE method was used to assess the association of each biometric parameter and the *HGF* SNP rs12536657 in the whole set of participants (without grouping), and also separately for case and control groups. Data from both eyes for each participant were used in this analysis, considering them not as independent values but taking into account the correlation between the two eyes of the same patient (analyzed as a pair) through the GEE method. GEE is an extension of linear regression that offers the advantage of keeping the data from both eyes for each participant while taking into account the correlation between the two eyes [44]. All regression tests were implemented using SPSS (version 20.0; SPSS Inc.), and *p* values <0.05 were considered statistically significant.

## 3. Results

A total of 403 individuals (194 males and 209 females), including 188 hyperopic children (5 to 17 years; SE: ≥+3.50 D), 52 emmetropic children (13 to 17 years; SE: >−0.50 D <+1.25 D), and 163 emmetropic young adults (18 to 28 years; SE: >−0.50 D <+1.25 D), were included in the analysis. There were significant differences in the proportions of men and women between the emmetropic and hyperopic groups, and therefore, all the subsequent analyses were adjusted for gender. The mean SE was +5.79 ± 1.47 D (+3.50 to +11.75) for the hyperopic group and +0.06 ± 0.45 D (−0.50 to +1.13) for the emmetropic groups. Reliable corneal topography could be achieved in 89.33% of the cases, and IOL master biometry was achieved in 95.21% of cases. Baseline refractive and biometric measurements for each group are summarized in Table 1.

Genotype frequencies of rs12536657 were in agreement with Hardy–Weinberg expectations in controls (*p*=0.12). Gene-condition associations were analyzed separately between all the emmetropic patients and the hyperopic children; between the emmetropic children group and the hyperopic children group; and between the emmetropic adult group and the hyperopic children. Genotypic tests did not support an association between hyperopia and rs12536657 in any of the groups: either among emmetropic and hyperopic patients (*p*=0.28, chi-squared test), nor among pediatric groups (*p*=0.41, chi-squared test), nor among hyperopic and adult emmetropic group (*p*=0.27, chi-squared test). Likewise, allelic tests yielded nonsignificant results for rs12536657 disease associations among the different groups (Supplementary Materials (available here)). Gender-adjusted logistic regression analyses did not detect significant associations of the genotypes of rs12536657 with hyperopia among children or adults (Table 2). Conditional logistic regression was performed between a set of exact age-matched case and controls to test if there was any association between age and genotypes, and no significant association was found (*p*=0.97, data not shown).

Since no differences in the distribution of the genotypes or alleles were found among any of the groups, a total of 403 pairs of eyes were included in the age- and gender-adjusted quantitative trait analysis. Significant univariate associations between mean topographic corneal curvature (*K*_mean_) and rs12536657 for the G/A (slope = +0.32; CI 95%: 0.04–0.60; *p*=0.023) and A/A (slope = +0.76; CI 95%: 0.12–1.40; *p*=0.020) genotypes were observed with the age- and gender-adjusted GEE model. Both flat and steep corneal topographic meridians were also significantly associated with rs12536657 for the G/A and A/A genotypes. An additive effect was observed in the corneal curvature in all topographic measurements with a mean difference increase between 0.31 D and 0.34 D for the G/A genotype and between 0.69 D and 0.76 D for the A/A genotype compared to the homozygous genotype G/G (Tables 3 and 4). As expected, when IOL master keratometric measurements were analyzed, significant associations were also observed between *K*_mean_ biometric corneal curvature and rs12536657 for the G/A (slope = +0.29; CI 95%: 0.02–0.56; *p*=0.033) and A/A (slope = +0.70; CI 95%: 0.08–3.75; *p*=0.026) genotypes. The G/A and A/A genotypes of rs12536657 were also significantly associated with steep corneal biometric meridian. A similar additive effect was also observed for the heterozygous and homozygous minor genotypes in reference to the homozygous major genotype. Therefore, based on the results of the univariate tests, patients with the A risk allele tended to have a steeper corneal curvature in both flat and steep meridians independently of the age and gender of the subject. However, topographic and biometric astigmatism were not associated with any of the genotypes. None of the other refractive or biometric measurements were significantly associated with the analyzed SNP. Multivariate association tests were not performed since corneal curvature was the unique significant variable found in the univariate analysis. Nevertheless, corneal curvature parameters would not remain statistically significant after multiple testing correction but showed a trend to significance. Similarly, when gender- and age-adjusted GEE was applied separately in cases and controls, corneal curvature remained as the only variable showing tendency towards a significant association with rs12536657 genotypes (*K*_mean_: *p*=0.07 in cases and *p*=0.08 in controls, age- and gender-adjusted GEE method). The presence of higher *p* values when analyses were performed separately in only case or control groups can be attributed to the sample size difference caused by the grouping.

## 4. Discussion

To our knowledge, this is the first quantitative trait association study of an *HGF* gene variant and ocular biometric parameters with a hyperopic pediatric, emmetropic adolescent, and young adult European Caucasian cohort. We selected *HGF* gene SNP rs12536657 that was previously reported as associated with hyperopia in an Australian adult population [24]. The most interesting findings we report are the trends towards significant associations that were observed for all corneal curvature measurements and the minor A allele of the rs12536657 of the *HGF* gene under an additive genetic model. Of note, these associations were detected in an age- and gender-independent manner, using two different measurement methods (biometry and topography) and including data from 806 eyes. We observed a mean increase in corneal curvature in both meridians of between 0.31 D and 0.34 D for the G/A genotype and between 0.69 D and 0.76 D for the A/A genotype compared to patients with the homozygous genotype G/G. Therefore, patients with the A allele in their genotype (either homozygous or heterozygous) tended to have a steeper corneal curvature. Corneal curvature and axial length are the main biometric measures (endophenotypes) that establish the refractive status of the eye. The cornea is the most important refracting element, and its curvature must be thoroughly coordinated with the dimensions of the other components of the growing eye during childhood. The heritability estimate for corneal curvature in the Beaver Dam Eye Study was as high as 95% [45]. It is interesting to note that a set of shared genetic variants is largely responsible for the relative scaling of corneal curvature and axial length [40], and a linear correlation between axial length and corneal curvature has been demonstrated. Shorter emmetropic eyes usually have more peaked corneas to counteract the impact of axial length on refraction in what is termed the “stabilizing factor” [12]. On the other hand, corneal astigmatism was not found to be associated with SNP rs12536657, similarly to what has been reported for other genetic variants which, in spite of being associated with corneal curvature in large GWAS [39], were not associated with astigmatism [41, 42]. This finding highlights the fact that while corneal curvature is an ocular dimension, corneal astigmatism is an eye disorder which probably involves a separate group of genes [46].

The *HGF* gene locus is linked to the control of normal variation in eye size in mice, and *HGF* is also a potent mitogen expressed in the cornea, retina, pigment epithelium, and choroid [27, 47]. It would seem likely that *HGF* has some role to play in corneal development and in the maintenance of normal structure in the adult cornea [48]. Regardless of its implication in several cellular roles within the cornea [28], *HGF* with its receptor c-MET is known to be expressed in the cornea in all three cellular layers [27]. Some gene variants for c-MET have shown a suggestive genetic association with corneal curvature although they did not remain statistically significant after multiple testing correction [49]. Several *HGF* variants have been associated with keratoconus [26, 27], and even increased *HGF* protein expression within corneal epithelium has been reported for keratoconic patients [28]. The association of the *HGF* gene and corneal curvature has been studied by Sahebjada et al. who analyzed 10 SNPs in the *HGF* gene in a case-control study on patients with keratoconus. However, they could not detect any significant association of the chosen SNPs with corneal curvature, although tag SNP rs2286194 was the closest to significance (*p*=0.049) [26]. Interestingly, Yanovitch et al. reported the same SNP as having a strong association with mild to moderate myopia [23]. In any case, the association of corneal curvature with the SNP rs12536657 that we have uncovered had not been previously assessed.

On the other hand, our results did not support the association of this *HGF* gene SNP with hyperopia for the Spanish pediatric population. The discrepancy between the results of our study and that of Veerappan et al. [24] may have occurred because our study focused on the Spanish Caucasian population, whereas Veerappan et al. performed their study on a population of Anglo-Celtic ethnicity. Since SNP alleles show significant geographical or ethnic group variations in human populations, it is likely that different SNPs may be associated with refraction in individuals from different ethnicities. Besides, a different study design was employed. Although the association of *HGF* variants with myopia had been previously reported and replicated in several independent studies on adults in Chinese [21] and Caucasian populations [23, 24], we were interested in studying children with high and moderate hyperopia. Recent studies point at age-specific effects of genetic variants associated with refractive error and ocular biometry. In fact, predisposing SNPs have been found to differ in the age at which effects are present and in whether or not these effects get progressively stronger during later childhood [50]. For example, in the case of myopia, a meta-analysis suggested that specific loci have their greatest effect in young children, while others reach the greatest effect during early teenage years [51]. These are some of the reasons why we carefully selected a group of moderate and high hyperopic children instead of a group of hyperopic adults.

The strengths of the current study include being the first multicenter study of genetic association in Spanish pediatric hyperopic individuals comprising a relatively large homogenous population of Caucasian ancestry. The cohort was Spanish Caucasian based on family history and ethnicity features. Since all the participants were from locations situated in nearby cities in the north and the centre of Spain, it is less probable that large genetic variations have stratified the population, thus affecting the results. In any case, genotype frequency comparisons between different hospital sites providing most of the participants of the study were compared, and no differences were found (*p*=0.18, chi-squared test). This allows for minimization of potential effects of population admixture and also addresses calls for more diversity in genetic studies [52, 53]. In addition, a study including cycloplegic refraction in a group of young emmetropic adults is uncommon [54]. Besides, corneal parameters have been extensively studied by topographic corneal analysis including most of the important corneal features and not only corneal curvature. Quantitative trait analyses take the full spectrum of measures into account and constitute a powerful tool to detect genetic contributions that has several advantages over case-control studies [55]. Since fewer genes are likely to impact endophenotypes such as axial length or corneal curvature when compared to the composite phenotype of quantitative refraction [5], examining these intermediate traits independently is likely to result in greater power to detect variants associated with these phenotypes [45]. In addition, for the present study, quantitative trait analysis was performed through GEE, an advanced statistical framework that addresses unknown correlation structures within the data. The primary interest in ophthalmic genetic studies for quantitative traits is often to locate genetic loci that exert effects on both eyes. The use of averaged ocular measurements has been the convention in the study of quantitative traits in the research community of refractive error genetics. However, the GEE method allows us to properly utilize information for both eyes and is considered to be more robust than to use the mean of the measurements of both eyes or the data of one randomly chosen eye [56].

At the same time, several limitations have to be recognized in our work. First, only one *HGF* gene variant has been analyzed in the present study, so very limited coverage of *HGF* gene information was achieved. Veerappan et al. performed tag SNP analysis and identified 9 tag SNPs that included most of the genetic information for the *HGF* gene; they also sequenced the coding regions of the *HGF* gene, and other 6 SNPs were identified [24]. Of the 15 *HGF*, SNPs analyzed in their cohort of emmetropes, hyperopes, and myopes, they found 6 SNPs associated with myopia (rs1743, rs4732402, rs12536657, rs10272030, rs9642131, and rs5745646, all in high linkage disequilibrium with each other) and 2 with hyperopia (rs5745718 and rs12536657, also in high LD with each other). They admitted that it was unclear how SNP rs12536657 could be associated with both hypermetropia and myopia, but it is important to highlight that Yanovitch et al. also detected an association of this SNP with myopia [23]. Second, we decided not to include a myopic group in order to focus our study on hyperopia as children are by far the target population that is most affected by the main complications of hyperopia. Myopia, in contrast, presents a much lower morbidity in childhood. Third, with respect to the case-control study, the difference in age between cases and controls represents a source of potential bias, and thus, we divided the gene-condition analysis considering the two separated emmetropic groups (children and adults). In addition to the fact that genotype does not change during the lifespan, change-in-estimate criterion analysis did not point to age as a confounder for study findings, and we obtained similar confirmation through logistic conditional regression analysis. Nevertheless, association tests were adjusted by age and gender when appropriate. Although we included a group of emmetropic children aged 13 to 17, we intentionally did not include any children aged between 5 and 13 years in this group because of the difficulty of having a stabilized emmetropic group of this age due to the growing eyes of these children [33]. In any case, a small proportion of the emmetropic children aged 13 to 17 may develop later onset myopia in the future, and thus, we also performed the analyses with a young adult emmetropic group. It is important to highlight that moderate and high hyperopia at these ages are considered to be much more stable through time with much less refractive changes. And last, even though the power calculation for this study suggests that the current cohort size per group (188 hyperopes and 215 emmetropes) is adequate to detect modest genetic effects up to an OR of 1.8, we cannot rule out the possibility that smaller effect sizes may have been missed. In any case, the size per group in our study is larger than that of the cohort of Veerappan et al. in which a positive association was shown for SNPs rs12536657 and rs5745718 and hyperopia [24].

Since the publication of the findings of Veerappan et al., other genetic variants have been associated with corneal curvature as well as with hyperopia in GWAS studies. Association of platelet-derived growth factor receptor alpha (PDGFRA) with corneal curvature has been identified in several different populations [38–40]. Simpson et al. reported two genome-wide significant associations with hyperopia for a case-control GWAS meta-analysis from 9 studies of European-derived adult populations. These regions overlapped with loci 15q14 (rs11073060) and 8q12 (rs10089517) [25]. The locus on 15q14 is within an intergenic region in the vicinity of the genes gap junction protein delta 2 (GJD2) and actin alpha cardiac muscle 1 (ACTC1), two genes that are expressed in the retina and are potential candidate genes for refractive error [6]. Jiang et al. identified several variants in the serine protease 56 (PRSS56) gene associated with high hyperopia [57]. In any case, refractive error and coordinated scaling of ocular component dimensions are such complex phenotypes influenced by so many common genetic variants and environmental factors that their study will remain challenging for years to come [58, 59].

## 5. Conclusions

In conclusion, although our findings only cover one facet of the complex polygenic nature of the studied phenotype, our results indicate a potential role for rs12536657 in corneal curvature in our population. To our knowledge, this is the first multicenter quantitative trait association study of an *HGF* SNP and ocular biometric parameters comprising a pediatric cohort. Further work studying variants in *HGF* and other reported cornea-related genes is warranted to confirm these findings for different ethnic groups.

## Figures and Tables

**Table 1:** Baseline refractive and biometric measurements for cases (hyperopic children) and controls (emmetropic children and emmetropic adults).

	Hyperopic children (*n*=188)		Emmetropic children (*n*=52)		Emmetropic adults (*n*=163)	
Age (years)	8.23 (2.62)		16.48 (1.18)		22.26 (2.55)	
Gender (female)	81 (43.1%)		25 (48.1%)		103 (63.2%)	
	Mean (SD)		Mean (SD)		Mean (SD)	
	RE	LE	RE	LE	RE	LE
SE	+5.71 (1.47)	+5.87 (1.46)	+0.07 (0.45)	+0.07 (0.46)	+0.05 (0.45)	+0.05 (0.44)
Sphere	+6.20 (1.57)	+6.39 (1.53)	+0.14 (0.45)	+0.14 (0.45)	+0.16 (0.47)	+0.19 (0.45)
Cylinder	−0.98 (0.88)	−1.05 (0.87)	−0.14 (0.24)	−0.15 (0.26)	−0.22 (0.25)	−0.28 (0.25)
*K*_flat_ TOP	41.99 (1.40)	42.03 (1.39)	42.54 (1.46)	42.62 (1.42)	43.30 (1.37)	43.43 (1.30)
*K*_steep_ TOP	43.54 (1.41)	43.61 (1.39)	43.12 (1.47)	43.35 (1.46)	43.93 (1.38)	44.13 (1.32)
*K*_mean_ TOP	42.76 (1.33)	42.82 (1.32)	42.83 (1.45)	42.87 (1.46)	43.61 (1.36)	43.69 (1.33)
ACD TOP	2.88 (0.28)	2.89 (0.28)	3.28 (0.31)	3.26 (0.30)	3.19 (0.25)	3.19 (0.25)
WW TOP	11.99 (0.39)	11.97 (0.38)	12.12 (0.37)	12.13 (0.36)	12.10 (0.37)	12.08 (0.38)
CCT TOP	564.39 (30.68)	564.81 (31.26)	542.48 (34.64)	543.02 (34.82)	542.01 (37.67)	543.48 (36.42)
AAA TOP	40.51 (6.92)	40.49 (8.00)	45.87 (6.82)	44.60 (8.31)	44.16 (6.16)	43.50 (6.61)
AL BIOM	21.20 (0.83)	21.11 (0.84)	23.59 (0.73)	23.55 (0.72)	23.40 (0.71)	23.35 (0.72)
*K*_flat_ BIOM	42.06 (1.39)	42.08 (1.41)	42.61 (1.45)	42.56 (1.45)	43.35(1.37)	43.36 (1.35)
*K*_steep_ BIOM	43.69 (1.44)	43.79 (1.42)	43.27 (1.44)	43.19 (1.48)	44.03(1.35)	44.01 (1.35)
*K*_mean_ BIOM	42.88 (1.33)	42.94 (1.34)	42.94 (1.44)	42.99 (1.43)	43.69(1.34)	43.78 (1.30)
ACD BIOM	3.28 (0.26)	3.28 (0.27)	3.61 (0.32)	3.62 (0.30)	3.62(0.25)	3.63 (0.24)
WW BIOM	12.19 (0.38)	12.16 (0.41)	12.20 (0.31)	12.23 (0.28)	12.08(0.56)	12.15 (0.41)

LE: left eye; RE: right eye; BIOM: IOL master measurements; TOP: topography measurements; SE: spherical equivalent (diopters); *K*_flat_: anterior flat keratometry (diopters); *K*_steep_: anterior steep keratometry (diopters); *K*_mean_: anterior mean keratometry (diopters); AL: axial length (mm); ACD: anterior chamber depth (mm); WW: white-to-white diameter (mm); CCT: central corneal thickness (microns); ACA: anterior chamber angle (degrees).

**Table 2:** Logistic regression gender-adjusted results for different genotypes of *HGF* SNP rs12536657 under an additive genetic model of hyperopic children compared with all emmetropic patients (a); hyperopic children compared with emmetropic children (b); and hyperopic children with emmetropic adults (c).

SNP rs5712536657	Genotype	Hyperopic children (*n*=185)^*∗*^	Emmetropic children and adults (*n*=214)^*∗∗*^	OR	95% CI for OR		*p*
(a)							0.206
	GG	116 (62.70)	135 (63.08)	1.00			
	GA	63 (34.05)	65 (30.37)	1.18	0.76	1.82	0.458
	AA	6 (3.24)	14 (6.54)	0.47	0.17	1.27	0.136

SNP rs12536657	Genotype	Hyperopic children (*n*=185)^*∗*^	Emmetropic children (*n*=52)	OR	95% CI for OR		*p*

(b)							0.30
	GG	116 (62.7%)	34 (65.4%)	1.00			
	GA	63 (34.1%)	14 (26.9%)	0.76	0.38	1.52	0.439
	AA	6 (3.2%)	4 (7.7%)	2.26	0.607	8.47	0.228

SNP rs12536657	Genotype	Hyperopic children (*n*=185)^*∗*^	Emmetropic adults (*n*=162)^*∗∗*^	OR	95% CI for OR		*p*

(c)							0.23
	GG	116 (62.7%)	101 (62.3%)	1.00			
	GA	63 (34.1%)	51 (31.5%)	1.16	0.73	1.86	0.531
	AA	6 (3.2%)	10 (6.2%)	0.45	0.15	1.30	0.140

^*∗*^Missing genotype data: in the hyperopic group, 3 genotypes were missing (*n*=185). ^*∗∗*^Missing genotype data: 1 genotype was missing in the emmetropic adult group (*n*=214 and *n*=162).

**Table 3:** Univariate generalized estimating equation method of topographic measurements with *HFG* rs12536657 under an additive genetic model adjusted for age and gender.

	Genotype rs12536657	Coef.	95% CI for slope		*p*
			Lower	Upper	
*K*_mean_ (TOP) (D)					**0.010**
	G/G (ref.)	41.88			
	G/A	0.32	0.04	0.60	**0.023**
	A/A	0.76	0.12	1.40	**0.020**

*K*_flat_ (TOP) (D)					**0.019**
	G/G (ref.)	40.89			
	G/A	0.31	0.02	0.60	**0.036**
	A/A	0.69	0.06	1.31	**0.031**

*K*_steep_ (TOP) (D)					**0.007**
	G/G (ref.)	42.87			
	G/A	0.34	0.05	0.63	**0.021**
	A/A	0.83	0.16	1.51	**0.015**

ACD (TOP) (mm)					0.787
	G/G (ref.)	2.80			
	G/A	0.00	−0.06	0.05	0.875
	A/A	−0.04	−0.16	0.07	0.489

WW (TOP) (mm)					0.424
	G/G (ref.)	11.98			
	G/A	−0.02	−0.10	0.07	0.703
	A/A	−0.13	−0.32	0.07	0.195

CCT (TOP) (*µ*m)					0.191
	G/G (ref.)	578.63			
	G/A	−6.39	−13.37	0.60	0.073
	A/A	0.04	0.04	17.45	0.996

Apical CT (TOP) (*µ*m)					0.812
	G/G (ref.)	610.27			
	G/A	−3.70	−14.98	7.58	0.520
	A/A	−0.98	−23.72	21.77	0.933

TOP: topography measurements; *K*_flat_: anterior flat keratometry (diopters); *K*_steep_: anterior steep keratometry (diopters); *K*_mean_: anterior mean keratometry; ACD: anterior chamber depth (mm); WW: white-to-white diameter (mm); CCT: central corneal thickness; apical CT: apical corneal thickness. For reference categories, coefficient presented is the intercept (mean of ref. category), and for other categories, it is the slope (mean difference).

**Table 4:** Univariate generalized estimating equation method to analyze association of refractive and biometric measurements with *HFG* rs12536657 under an additive genetic model adjusted for age and gender.

	Genotype rs12536657	Coef	95% CI for slope		*p*
			Lower	Upper	
SE (D)					0.930
	G/G (ref.)	8.50			
	G/A	0.04	−0.27	0.34	0.817
	A/A	−0.07	−0.64	0.49	0.796

Cyl (D)					0.857
	G/G (ref.)	−1.38			
	G/A	0.01	−0.12	0.14	0.885
	A/A	−0.05	−0.26	0.15	0.618

AL (BIOM) (mm)					0.236
	G/G (ref.)	20.29			
	G/A	−0.12	−0.28	0.05	0.166
	A/A	−0.22	−0.58	0.14	0.231

*K*_mean_ (BIOM) (D)					**0.016**
	G/G (ref.)	42.11			
	G/A	0.29	0.02	0.56	**0.033**
	A/A	0.70	0.08	3.75	**0.026**

*K*_flat_ (BIOM) (D)					**0.037**
	G/G (ref.)	41.04			
	G/A	0.31	−0.01	0.55	0.055
	A/A	0.63	0.00	1.25	**0.049**

*K*_steep_ (BIOM) (D)					**0.009**
	G/G (ref.)	43.17			
	G/A	0.31	0.03	0.59	**0.027**
	A/A	0.78	0.15	1.41	**0.015**

ACD (BIOM) (mm)					0.319
	G/G (ref.)	3.15			
	G/A	−0.01	−0.07	0.04	0.680
	A/A	−0.08	−0.19	0.02	0.132

SE: spherical equivalent; Cyl: cylinder; BIOM: IOL master measurements; AL: axial length (mm); *K*_flat_: anterior flat keratometry (diopters); *K*_steep_: anterior steep keratometry (diopters); *K*_mean_: anterior mean keratometry; ACD: anterior chamber depth (mm). For reference categories, coefficient presented is the intercept (mean of ref. category), and for other categories, it is the slope (mean difference).

## Data Availability

The data used to support the findings of this study are available from the corresponding author upon request.

## Supplementary Materials

Supplementary Materials Genotypic and allelic test hyperopic children versus emmetropic children and hyperopic children versus emmetropic adults.
