# Supplementary material for: HGF-rs12536657 and Ocular Biometric Parameters in Hyperopic Children, Emmetropic Adolescents, and Young Adults: A Multicenter Quantitative Trait Study
Source: J Ophthalmol. 2019 Feb 3;2019:7454250. doi: 10.1155/2019/7454250 (PMC6378066; doi:10.1155/2019/7454250)
Supplement: Supplementary Materials — Genotypic and allelic test hyperopic children versus emmetropic children and hyperopic children versus emmetropic adults. [file 7454250.f1.pdf]

Suplemmentary material

Genotypic and allelic test hyperopic children versus all emmetropic patients (chi squared test)

SNP rs12536657		Hyperopic children	Emmetropic children and adults	p (chi^2)*
Genotype	GG	116 (62.7%)	135 (63.1%)	0.28
	GA	63 (34.1%)	65 (30.4%)	
	AA	6 (3.2%)	14 (6.5%)	
	Total	185 (100%)	214 (100%)	

Allele		Hyperopic children	Emmetropic children and adults	p (chi^2)*
	G	295 (79.7%)	335 (78.3%)	0.66
	A	75 (20.3%)	93 (21.7%)	
	Total	370 (100%)	428 (100%)	

Genotypic and allelic test hyperopic children versus emmetropic children (chi squared test)

SNP rs12536657		Hyperopic children	Emmetropic children	p (chi2)
Genotype	GG	116 (62.7%)	34 (65.4%)	0.27
	GA	63 (34.1%)	14 (26.9%)	
	AA	6 (3.2%)	4 (7.7%)	
	Total	185 (100%)	52 (100%)	

Allele		Hyperopic children	Emmetropic children	p (chi2)
	G	295 (79.7%)	82 (78.8%)	0.84
	A	75 (20.3%)	22 (21.2%)	
	Total	370 (100%)	104 (100%)	

Genotypic and allelic test hyperopic children versus emmetropic adults (chi squared test)

SNP rs12536657		Hyperopic children	Emmetropic adults	p (chi2)
Genotype	GG	116 (62.7%)	101 (62.3%)	0.28
	GA	63 (34.1%)	51 (31.5%)	
	AA	6 (3.2%)	10 (6.2%)	
	Total	185 (100%)	162 (100%)	

Allele		Hyperopic children	Emmetropic adults	p (chi2)
	G	295 (79.7%)	253 (78.1%)	0.59
	A	75 (20.3%)	71 (21.9%)	
	Total	370 (100%)	324 (100%)	
